# Arterial Pressure and Flow Wave Analysis Using Time-Domain 1-D Hemodynamics

**DOI:** 10.1007/s10439-014-1087-4

**Published:** 2014-08-20

**Authors:** Marie Willemet, Jordi Alastruey

**Affiliations:** Division of Imaging Sciences and Biomedical Engineering, St. Thomas’ Hospital, King’s College London, London, UK

**Keywords:** Pulse wave propagation, Wave intensity analysis, Peripheral wave reflections, Reservoir pressure, Windkessel function

## Abstract

We reviewed existing methods for analyzing, in the time domain, physical mechanisms underlying the patterns of blood pressure and flow waveforms in the arterial system. These are wave intensity analysis and separations into several types of waveforms: (i) forward- and backward-traveling, (ii) peripheral and conduit, or (iii) reservoir and excess. We assessed the physical information provided by each method and showed how to combine existing methods in order to quantify contributions to numerically generated waveforms from previous cardiac cycles and from specific regions and properties of the numerical domain: the aortic root, arterial bifurcations and tapered vessels, peripheral reflection sites, and the Windkessel function of the aorta. We illustrated our results with numerical examples involving generalized arterial stiffening in a distributed one-dimensional model or localized changes in the model parameters due to a femoral stenosis, carotid stent or abdominal aortic aneurysm.

**Electronic supplementary material:**

The online version of this article (doi:10.1007/s10439-014-1087-4) contains supplementary material, which is available to authorized users.

## Introduction

The shape of arterial blood pressure and flow waves is determined by physical properties of the cardiovascular system, such as vessel geometry and stiffness, which can be altered by medical conditions (e.g., arteriosclerosis and hypertension) and lead to considerable variations in pressure and flow waveforms.^21^ Understanding how pressure and flow waveforms relate to cardiovascular properties is important for identifying specific drug targets for pharmacological treatment^17^ and deriving non-invasive diagnostic methods; e.g., arterial stiffness is an important predictor of cardiovascular events which cannot be directly measured in the clinic but can be calculated by wave analysis.^37^

One-dimensional (1-D) modeling is commonly used to contribute to this understanding. It enables us to simulate arterial pressure and flow waveforms by accounting for the distributed geometrical and mechanical properties of the arterial tree, ejection characteristics of the ventricle, and resistance of smaller arteries and arterioles. Several comparisons against *in vivo*,^22^,^27^,^32^,^34^,^40^ *in vitro* 4,9,13,29,30 and 3-D numerical^41^ data have shown the ability of the 1-D formulation to capture the main features of pressure and flow waveforms in human systemic arteries. Using 1-D modeling we can modify any model parameters (e.g., aortic stiffness, peripheral resistances) at will to investigate their effects on pressure and flow waveforms with reasonable computational cost. This is difficult to achieve *in vivo* for technical and physiological reasons, such as the inaccessibility of many of the vessels and the inability to isolate variables without compensatory effects of cardiovascular homeostatic reflexes.

Many studies have modeled arterial pressure and flow waveforms using the 1-D formulation, for instance to investigate physical mechanisms underlying hypertension^28^ and other medical conditions,^19^ as well as the outcome of surgical interventions.^13^,^18^,^32^,^40^ Fewer studies, however, have post-processed the 1-D model results by separating the simulated waveforms into components originating at specific regions of the 1-D domain, such as the aortic root, arterial bifurcations and peripheral reflection sites, to elucidate key physical mechanisms.

Several methods of wave analysis have been proposed, both in the frequency and time domains, and mainly applied to investigate *in vivo* data. Frequency-domain methods usually assume waveforms to be periodic, which is a limitation since real data are not perfectly periodic and may contain transient signals (e.g., due to flow control mechanisms). On the other hand, time-domain methods allow us to study non-periodic and transient waveforms and may be easier to understand by clinicians. They include wave intensity analysis,^14^,^17^,^24^ separation of pressure and flow waveforms into forward and backward components,^10^,^25^ and separation of the pressure waveform into reservoir and excess components.^11^,^38^ These will be described in detail and studied here.

The aim of this study is to assess the strengths and weaknesses of existing methods for analyzing, in the time domain, physical mechanisms underlying the pattern of arterial pressure and flow waveforms. We also propose a new method that improves wave analysis by combining the strengths of existing methods. We illustrate our results using waveforms generated by a 1-D model in the aorta, carotid, brachial and iliac arteries, under normal physiological conditions, with generalized stiffening of the arterial wall, or with the presence of a single stenosis, stent or aneurysm. We chose these arteries because clinical measurements in them are feasible and relevant.

## Methods

We describe the 1-D formulation (“Arterial 1-D Formulation” section), distributed 1-D model (“The Studied Arterial 1-D Models” section) and methods of wave analysis (“Pulse Wave Analysis Methods” and “Combined Pulse Wave Analysis” sections) that will be studied.

### Arterial 1-D Formulation

In the 1-D formulation the arterial network is decomposed into arterial segments connected to each other at nodes. Each segment is modeled as a deformable tube whose properties can be described by a single axial coordinate *x*. Under the assumption of incompressible and Newtonian fluid, the 1-D governing equations of blood flow can be derived from applying conservation of mass and momentum in an impermeable control volume of the arterial segment,^26^

1a $$\begin{aligned} \frac{\partial A}{\partial t} + \frac{\partial \left( AU \right) }{\partial x} = 0,\end{aligned}$$

1bwhere *t* is the time, *A*(*x*,*t*) is the cross-sectional area of the lumen, *U*(*x*,*t*) is the axial blood flow velocity averaged over the cross-section, *P*(*x*,*t*) is the blood pressure averaged over the cross-section, *ρ* = 1050 kg m^−3^ is the density of blood, and *f*(*x*,*t*) = −2*πμ*(*γ* + 2)*U* is the frictional force per unit length, with *γ* a constant parameter that depends on the shape of the velocity profile and *μ* = 4 mPa s the viscosity of blood. According to Smith *et al*.,^31^ *γ* = 9 corresponds to a velocity profile close to plug flow and is a good assumption for large arteries. This leads to *f*(*x*,*t*) = −22*πμU*. The terms in the conservation of momentum are the temporal acceleration (TA), convective acceleration (CA), pressure gradient force per unit mass (PG), and viscous force per unit mass (VF).

An explicit algebraic relationship between *P* and *A* (or *tube law*) is required to account for the fluid-structure interaction part of the problem and close equations (1). Here we model the arterial wall as a thin, incompressible, homogeneous, isotropic, elastic membrane characterized by an elastic modulus *E*(*x*) and thickness *h*(*x*). Under these premises we have^12^

2 $$\begin{aligned} P = P_{{\text{d}}}+\frac{\beta }{A_{{\text{d}}}} \left( \sqrt{A} - \sqrt{A_{{\text{d}}}} \right) , \end{aligned}$$

where *A*_d_(*x*) is the luminal area at diastolic pressure *P*_d_ and $\beta (x) = \frac{4}{3}\sqrt{\pi } E h$ accounts for the material properties of the arterial wall.

#### Characteristics Analysis—The Pulse Wave

Equations (1a), (1b), and (2) form a system of hyperbolic partial differential equations that can be analyzed using Riemann’s method of characteristics.^25^ For any point (*X*, *T*) in the (*x*, *t*) space there are two characteristic paths, *C*_f_ and *C*_b_ defined by $C_{\text{f,b}} \equiv \frac{{\text{d}} \hat{x}_{\text{f,b}}}{{\text{d}} t} = U \pm c,$ on which the *characteristic variables* *W*_f_ and *W*_b_ satisfy^6^

3 $$\begin{aligned} \frac{{\text{d}} W_{\text{f,b}}(\hat{x}_{\text{f,b}}(t),t)}{{\text{d}} t}= \frac{1}{\rho }\left( \frac{f}{A}- \frac{\partial P}{\partial \beta } \frac{{\text{d}} \beta }{{\text{d}} x}- \frac{\partial P}{\partial A_{{\text{d}}}} \frac{{\text{d}} A_{{\text{d}}}}{{\text{d}} x}\right) \end{aligned}$$

and

4 $$\begin{aligned} c = \sqrt{\frac{A}{\rho } \frac{\partial P}{\partial A}}= \sqrt{\frac{\beta }{2 \rho A_{{\text{d}}}}} A^{1/4}. \end{aligned}$$

If we further assume locally that fluid viscous losses are negligible and vessel properties are uniform, then^25^

5a $$\begin{aligned} \frac{{\text{d}} W_{\text{f,b}}(\hat{x}_{\text{f,b}}(t),t)}{{\text{d}} t} = 0, \end{aligned}$$

5b $$\begin{aligned} {\text{d}} W_{\text{f,b}} = {\text{d}}U \pm \frac{{\text{d}}P}{\rho c}{\quad {\text{on}} \quad \frac{{\text{d}} \hat{x}_{\text{f,b}}}{{\text{d}} t} = U \pm c}, \end{aligned}$$

which shows that d*W*_f,b_ are invariant along the characteristic paths and related to infinitesimal changes in *P* and *U*. Hereinafter, we will call d*P* and d*U* pressure and flow *wavefronts*, respectively. The term ‘wave’ refers to a change in blood pressure, flow and luminal area with a finite duration; ‘wavefront’ refers to infinitesimal changes in these quantities; and ‘waveform’ or ‘contour’ refers to the shape of these quantities over the cardiac cycle.

Under physiological flow conditions, *c* is much greater than the maximum *U* so that *U* + *c* > 0 and *U* − *c* < 0 (i.e., the flow is subcritical). As a result, d*P* and d*U* propagate in the forward and backward directions (we define the forward direction as the direction of mean blood flow, in which *x* increases) with speeds of *U* + *c* and *U* − *c* respectively; *c* is the speed at which wavefronts travel in the absence of convective velocity (*U*), which is referred to as *pulse wave velocity*. The *pulse* (the regular beating of arteries that follows cardiac contraction) is produced by changes in blood pressure (in our model *P* changes *A* through Eq. (2)) and, according to the characteristics analysis, propagates in the form of waves, referred to as *pulse waves*, running forwards and backwards.

### The Studied Arterial 1-D Models

**Figure 1:**
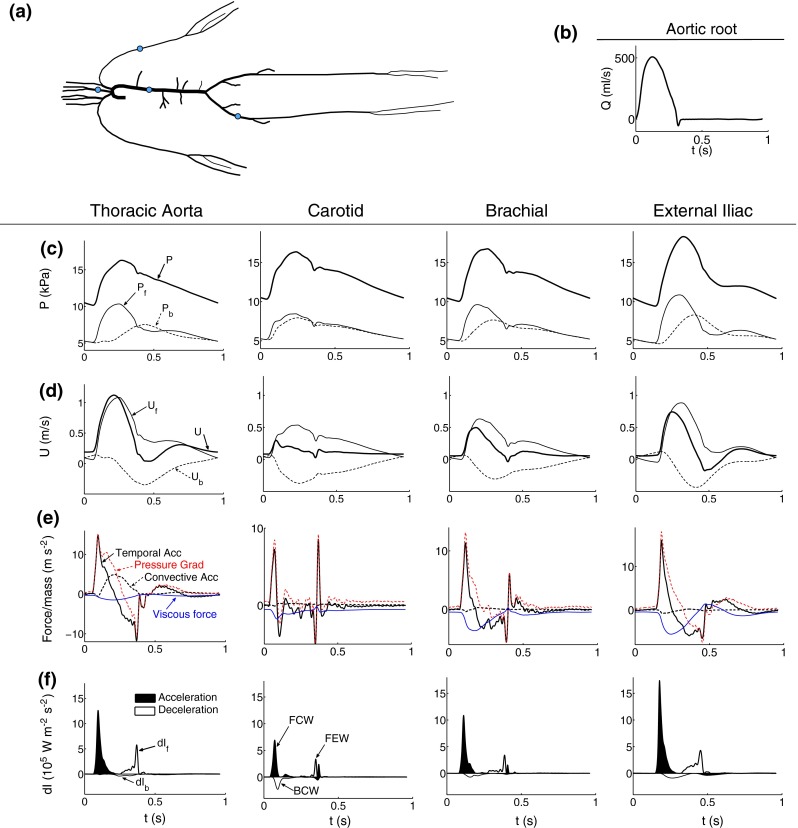
Pressure and velocity waveforms in the midpoint of the thoracic aorta, left carotid, left brachial, and right external iliac of the 55-artery model sketched in (a). The flow waveform prescribed at the inlet of the aortic root is shown in (b). (c) Pressure waveform (*P*) and its forward (*P*_f_) and backward (*P*_b_) components. (d) Velocity waveform (*U*) and its forward (*U*_f_) and backward (*U*_b_) components. (e) Components of the equation of momentum (temporal and convective accelerations, and pressure gradient and viscous forces) expressed as force per unit mass as shown in Eq. (1b). (f) Forward (d*I*_f_) and backward (d*I*_b_) components of wave intensity (d*I*). Shaded waves (black) accelerate blood flow and non-shaded waves (white) decelerate blood flow. Three significant waves are observed in each artery: a forward compression wave (FCW), backward compression wave (BCW) and forward expansion wave (FEW)

We generated all pressure and flow waveforms by solving the system of Equations (1a), (1b), and (2) in a model of the 55 larger systemic arteries in the healthy human (Fig. 1a). The flow waveform shown in Fig. 1b was prescribed at the aortic root as a reflective boundary condition, energy losses were neglected at bifurcations and boundary conditions, and terminal branches were coupled to three-element Windkessel models consisting of two peripheral resistances (*R*_1_ and *R*_2_) and one compliance (*C*). The initial conditions were (*A*,*U*,*P*) = (*A*_0_(*x*),0,0) in all segments, where *A*_0_ is the luminal area that yields *A*_d_ at *P* = *P*_d_.^41^ We refer the reader to Alastruey *et al*.^6^ for a detailed description of this model and the values of its parameters. Details on the numerical scheme are given in Refs. 4, 6.

**Figure 2:**
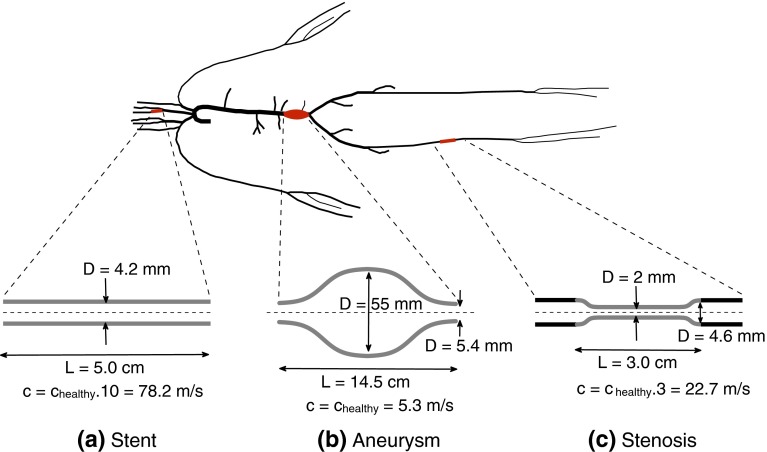
Sketch of the models of the (a) carotid stent, (b) abdominal aortic aneurysm, and (c) femoral stenosis used in this study. The stent starts at the inlet of the left internal carotid artery and has a constant diameter. The aneurysm extends over the last 15 cm of the abdominal aorta and its shape is based on Swillens *et al*.^35^ The stenosis is located in the middle of the right femoral artery and its properties are based on Li *et al*.^16^ *c*_healthy_: pulse wave velocity in the ‘healthy’ model

The parameters of the model described above—hereafter called the *‘healthy’* model—were changed to simulate four common clinical scenarios: (i) generalized arterial stiffening representative of an old subject^7^ and obtained by increasing *c* in all arterial segments by a factor of 1.5 (hereafter referred to as the *‘old’* model); (ii) a stent in the proximal left internal carotid (localized increase in stiffness; Fig. 2a); (iii) a fusiform aneurysm in the abdominal aorta (significant increase in the luminal area; Fig. 2b); and (iv) a stenosis in the medial right femoral artery (partial occlusion of the luminal area and localized increase in stiffness; Fig. 2c).

### Pulse Wave Analysis Methods

We assess the physical information provided by the following existing methods of pulse wave analysis. We used customized MATLAB software (The MathWorks, Inc., MA, USA) for all data analysis.

#### Wave Intensity Analysis (WIA)

*In vivo* measurements of *P* and *U* with time are typically taken at a fixed point rather than along a characteristic line. Thus, solving the two equations in Eq. (5b) at *x* = *X* for d*P*(*t*) and d*U*(*t*) yields

6 $$\begin{aligned} {\text{d}} P = \frac{\rho c}{2} \left( {\text{d}}W_{\text{f}} - {\text{d}}W_{\text{b}} \right) ,\quad {\text{d}} U = \frac{1}{2} \left( {\text{d}}W_{\text{f}} + {\text{d}}W_{\text{b}} \right) . \end{aligned}$$

*Wave intensity* (d*I*) is defined as^24^,^25^

7 $$\begin{aligned} {\text{d}} I = {\text{d}}P \, {\text{d}}U= \frac{\rho c}{4} \left( ({\text{d}}W_{\text{f}})^2 - ({\text{d}}W_{\text{b}})^2 \right) , \end{aligned}$$

which is the flux of energy per unit area carried by wavefronts as they propagate through *x* = *X*. d*I* has dimensions of power per unit area and is positive if d*W*_f_ > d*W*_b_ and negative if d*W*_f_ < d*W*_b_. Therefore, d*I* ‘measures’ the importance with time of changes in *P* and *U* in the forward and backward directions at *x* = *X*. As summarized in Table 1, whenever d*I* > 0, forward changes in *P* and *U* dominate over backward changes; the flow is accelerated if d*P* > 0 and decelerated if d*P* < 0. Whenever d*I* < 0, backward changes in *P* and *U* dominate; the flow is accelerated if d*P* < 0 and decelerated if d*P* > 0.

**Table 1:** Classification of d*I* waves as a function of the signs of d*P* and d*U*

	Acceleration, d*U* > 0	Deceleration, d*U* < 0
Compression, d*P* > 0	Forward Compression (FCW) d*I* > 0	Backward Compression (BCW) d*I* < 0
Expansion, d*P* < 0	Backward Expansion (BEW) d*I* < 0	Forward Expansion (FEW) d*I* > 0

In this study, the sampling frequency of all numerical waveforms is 1 kHz. Moreover, we normalized d*I* by d*t* ^2^ to make the magnitude of d*I* independent of the sampling frequency.

#### Forward- and Backward-Traveling Waveforms

The waveforms $P(t)$ and $U(t)$ at a fixed point $x=X$ can be separated into forward-traveling ($P_{\text{f}}(t),\,U_{\text{f}}(t)$) and backward-traveling ($P_{\text{b}}(t),\,U_{\text{b}}(t)$) components; i.e., $P=P_{\text{f}}+P_{\text{b}}$ and $U=U_{\text{f}}+U_{\text{b}}.$ We start by separating ${\text{d}}P$ and ${\text{d}}U$ into changes across the forward (${\text{d}}P_{\text{f}},\,{\text{d}}U_{\text{f}}$) and backward (${\text{d}}P_{\text{b}},\,{\text{d}}U_{\text{b}}$) wavefronts; i.e., ${\text{d}}P = {\text{d}}P_{\text{f}} + {\text{d}}P_{\text{b}}$ and ${\text{d}}U = {\text{d}}U_{\text{f}} + {\text{d}}U_{\text{b}}.$ Combining these two expressions with the *water hammer* equations, ${\text{d}}P_{\text{f,b}} = \pm \rho c \, {\text{d}}U_{\text{f,b}},$ yields^25^

8 $$\begin{aligned} {\text{d}}P_{\text{f,b}} =\frac{1}{2} \left( {\text{d}}P \pm \, \rho c \, {\text{d}}U \right) ,\quad {\text{d}}U_{\text{f,b}} =\frac{1}{2} \left( {\text{d}}U \pm \, \frac{{\text{d}}P}{\rho c} \right) . \end{aligned}$$

Wave intensity at a fixed point $x=X$ can then be separated into forward (${\text{d}}I_{\text{f}} > 0$) and backward (${\text{d}}I_{\text{b}} < 0$) components using

9 $$\begin{aligned} {\text{d}}I_{\text{f,b}} = {\text{d}}P_{\text{f,b}} \, {\text{d}}U_{\text{f,b}}= \frac{\pm 1}{4 \rho c} \left( {\text{d}}P \pm \, \rho c \, {\text{d}}U \right) ^2. \end{aligned}$$

The water hammer equations assume locally the flow to be inviscid and the artery to have uniform properties. They can be derived from the system of equations Eqs. (1a) and (1b) using the method of characteristics^25^ or by applying directly conservation of mass and momentum to a control volume moving with the forward or backward pulse wavefronts (Ref. 3, Appendix 1).

Numerically, the time-varying pulse wave velocity ($c$) is known for each time step and, hence, Eq. (8) allows us to obtain $P_{\text{f,b}}(t)$ and $U_{\text{f,b}}(t)$ from the measured $P(t)$ and $U(t)$ by adding the differences ${\text{d}}P_{\text{f,b}}(t)$ and ${\text{d}}U_{\text{f,b}}(t)$; i.e., $P_{\text{f,b}}(t) = \sum {\text{d}}P_{\text{f,b}}(t) + P_0$ and $U_{\text{f,b}}(t) = \sum {\text{d}}U_{\text{f,b}}(t) + U_0.$ The integration constants $P_0$ and $U_0$ were taken to be half the pressure and velocity, respectively, at the end of diastole.

Equations (8) and (9) are used in most of the practical applications of WIA. They are a linearized versions of the ‘exact’ solution, since they assume forward and backward waves to be additive, waves to travel at $\pm c$ instead of $U \pm c,$ and $c$ not to be a function of pressure. A fully nonlinear separation of forward- and backward-traveling waves is given in Stergiopulos *et al*.^33^

#### Conduit and Peripheral Waveforms

Neglecting nonlinear effects we can separate the pressure, flow and velocity waveforms at an arbitrary point in an arterial network simulated using the 1-D formulation into a *conduit waveform*, ($P_{\text{con}},\,Q_{\text{con}},\,U_{\text{con}}$), which is made up of wavefronts propagating from the aortic root and their reflections at arterial junctions, tapered vessels, and the aortic root, and a *peripheral waveform*, ($P_{\text{per}},\,Q_{\text{per}},\,U_{\text{per}}$), which is made up of wavefronts originating from reflections at the outlet of the terminal branches of the given network. As detailed in Alastruey *et al*.,^5^ the conduit waveform is obtained by running the 1-D simulation with each terminal branch coupled to a single resistance equal to the characteristic impedance at the outlet of the branch, so that any wavefront leaving the branch is completely absorbed by the boundary condition. In addition, we used luminal areas at diastolic pressure as initial conditions; i.e., $A(x,0)=A_{{\text{d}}}$ in all segments. The peripheral waveform is calculated as the difference between the total and conduit waveforms.

#### Reservoir and Excess Pressure Waveforms

Under normal physiological conditions, the pressure waveform ($P(x,t)$) in large systemic arteries is approximately uniform in space during approximately the last two thirds of diastole.^1^,^38^ Based on this observation, several studies have separated the pressure waveform into a *reservoir* component that describes well the diastolic decay in pressure and an *excess* component that contributes to ($P(x,t)$) mainly in systole. Several methods for calculating the reservoir and excess components have been proposed.^1^,^2^,^38^ Following the conclusions in Alastruey,^2^ Mynard *et al*.,^20^ and Vermeersch *et al*.^36^ we consider the following two methods for this study.

*The two-element Windkessel model* was used by Wang *et al.* 38 to calculate a space-independent reservoir pressure which is labeled $P_{\text{r,W2}}$ in this paper. Starting with the 1-D equations (1a), (1b), and (2) in a network with terminal branches coupled to three-element Windkessel models (as described in “The Studied Arterial 1-D Models” section), $P_{\text{r,W2}}$ can be related to distributed 1-D model parameters. This is achieved by neglecting nonlinearities, flow inertia and blood viscosity in all 1-D model arterial segments, and by assuming that wall compliance and fluid peripheral resistance are the dominant effects. Under these assumptions, $P_{\text{r,W2}}$ is given by Alastruey *et al*.^5^

10 $$P_{\text{r,W2}} = P_{\text{out}} + (P_{\text{in}}(T_0)-P_{\text{out}})e^{-\frac{t-T_0}{R_{\text{T}} C_{\text{T}}}} + \frac{e^{-\frac{t}{R_{\text{T}} C_{\text{T}}}}}{C_{\text{T}}} {\displaystyle \mathop\int\limits_{T_0}^t}\left( Q_{\text{in}}(t')+ \sum ^M_{j=2} \frac{C^j R^j_1 R^j_2}{R^j_2 + R^j_1}\frac{{\text{d}} q^j_{\text{out}} (t') }{{\text{d}} t'}\right) e^{\frac{t'}{R_{\text{T}} C_{\text{T}}}}dt', \quad t \ge T_0, $$

where $P_{\text{out}}$ is the constant outflow pressure at each terminal branch, $P_{\text{in}}(t)$ and $Q_{\text{in}}(t)$ are the pressure and flow waveforms at the aortic root, $T_0$ is the initial time of $P_{\text{in}}(t)$ and $Q_{\text{in}}(t),\,q^j_{\text{out}}(t)$ is the outflow in the 1-D model terminal segment $j$ ($j=2, \ldots , M,$ where $j=1$ is the inlet and $M-1$ is the number of terminal branches), and $N$ is the total number of arterial segments in the 1-D domain. The resistance at the aortic root ($R_{\text{T}}$) is computed as

11 $$\begin{aligned} R_{\text{T}} = \frac{ \overline{P_{\text{in}}} - P_{\text{out}}}{\text{CO}}, \end{aligned}$$

with $\overline{P_{\text{in}}}$ the mean blood pressure at the aortic root and CO the cardiac output. The total compliance of the 1-D model network ($C_{\text{T}}$) is equal to the sum of the total arterial conduit compliance ($C_{\text{c}}$) and total arterial peripheral compliance ($C_{\text{p}}$),

12 $$\begin{aligned} C_{\text{T}} = C_{\text{c}} + C_{\text{p}},\quad {C_{\text{c}} = \sum _{i=1}^N C^i_{\text{seg}}},\quad C_{\text{p}} = \sum _{j=2}^M \frac{R^j_2 C^j}{R^j_1 + R^j_2}, \end{aligned}$$

with $C_{\text{seg}}$ the compliance of each 1-D model segment, which is given by

13 $$\begin{aligned} C_{\text{seg}} = \frac{\overline{A_{\text{m}}} L}{\rho (\overline{c_{\text{m}}})^2},\quad \overline{A_{\text{m}}} = \frac{1}{L} \mathop\int\limits_0^L A_{\text{m}}(x) dx,\quad \overline{c_{\text{m}}} = \frac{1}{L} \mathop\int\limits_0^L c_{\text{m}}(x) dx, \end{aligned}$$

where $L$ is the vessel length, $A_{\text{m}}(x)$ and $c_{\text{m}}(x)$ are, respectively, the area and wave speed computed at mean blood pressure. We calculate the excess pressure ($P_{\text{e,W2}}(x,t)$) associated with $P_{\text{r,W2}}$ as $P_{\text{e,W2}} = P- P_{\text{r,W2}}.$

It is important to note that $P_{\text{r,W2}}$ is the pressure to which $P(x,t)$ tends when wave activity approaches zero in late diastole, since $P_{\text{r,W2}}$ assumes zero flow inertia.^2^ The pressure gradient required to drive the 1-D model flow during this period is provided by the relaxation of all compliant vessels. Moreover, even though $P_{\text{r,W2}}$ depends on the parameters of all outflow three-element Windkessel models, it gets closer to the two-element Windkessel pressure with increasing number of 1-D model segments that describe the arterial network. Indeed, for a given total compliance $C_{\text{T}},$ the ratio $C_{\text{c}}$ to $C_{\text{p}}$ increases with increasing number of arterial segments simulated as 1-D model vessels, so that $C^j$ ($j=2, \ldots , M$) decreases. As a result, the term $\sum ^M_{j=2} \frac{C^j R^j_1 R^j_2}{R^j_2 + R^j_1} \frac{{\text{d}} q^j_{\text{out}} (t') }{{\text{d}} t'}$ in Eq. (10) vanishes with increasing number of arterial segments. In the limit $C^j=0$ ($j=2, \ldots , M$), $P_{\text{r,W2}}$ is equal to the classic two-element Windkessel pressure used in Wang *et al*.^38^

*The three-element Windkessel model* was proposed by Westerhof *et al.* 39 as a model of the whole arterial system for pumping hearts. It consists of two resistances and a compliance, with the resistance at the outflow of the heart equal to the characteristic impedance of the ascending aorta ($Z_{\text{Ao}}$). This model allows us to calculate excess ($P_{\text{e,W3}}(x,t)$) and reservoir ($P_{\text{r,W3}}(x,t)$) pressures at an arbitrary arterial site as^2^

14 $$\begin{aligned} P_{\text{e,W3}} = Z_{\text{Ao}}Q_{\text{in}},\quad P_{\text{r,W3}} = P - Z_{\text{Ao}}Q_{\text{in}}, \end{aligned}$$

with the feet of $Q_{\text{in}}(t)$ time-aligned with the local pressure $P(x,t).$ Note that $P_{\text{r,W3}}$ is the pressure at the compliance element. As detailed in Alastruey *et al*.,^2^ $P_{\text{e,W3}}$ and $P_{\text{r,W3}}$ have the following physical meaning: at an arbitrary location in a 1-D model network with straight arterial segments and all arterial junctions well-matched for the propagation of forward-traveling wavefronts, $P_{\text{e,W3}}$ and $P_{\text{r,W3}}$ are respectively equivalent to the conduit and peripheral pressures introduced in “Conduit and Peripheral Waveforms” section, if nonlinear effects are neglected. $P_{\text{r,W3}}$ is made up of wavefronts originating from reflections at the outlet of the terminal branches of the given network, while $P_{\text{e,W3}}$ is made up of forward-traveling wavefronts propagated by the left-ventricular flow ejection.

### Combined Pulse Wave Analysis

We propose a novel method to analyze a given cardiac cycle of the pressure, $P(x,t),$ and flow rate, $Q(x,t),$ at an arbitrary point in a 1-D model network which combines all the methods described above. First the space-independent *history pressure waveform*, $P_{\text{his,1}}(t),$ is calculated by prolonging the diastolic decay in $P_{\text{r,W2}}$ from the previous cardiac cycle into the current cycle. Mathematically, $P_{\text{his,1}}$ is obtained from $P_{\text{r,W2}}$ (Eq. (10)) by taking $T_0$ to be the time at the start of cardiac ejection ($T_{\text{c}}$) and setting $Q_{\text{in}}=0$ for $t>T_{\text{c}}$ ($q^j_{\text{out}},\,j=2, \ldots , M,$ goes to zero with increasing $t>T_{\text{c}}$); i.e.,

15 $$P_{\text{his,1}} = P_{\text{out}}+ (P_{\text{in}}(T_{\text{c}})-P_{\text{out}})e^{-\frac{t-T_{\text{c}}}{R_{\text{T}} C_{\text{T}}}}, + \frac{e^{-\frac{t}{R_{\text{T}} C_{\text{T}}}}}{C_{\text{T}}} {\displaystyle \mathop\int\limits_{T_{\text{c}}}^t}\left( \sum ^M_{j=2} \frac{C^j R^j_1 R^j_2}{R^j_2 + R^j_1}\frac{{\text{d}} q^j_{\text{out}} (t') }{{\text{d}} t'}\right) e^{\frac{t'}{R_{\text{T}} C_{\text{T}}}}dt',\quad t \ge T_{\text{c}}.$$

Thus, $P_{\text{his,1}}$ is made up only of wavefronts generated in previous cardiac cycles.^3^ The *cycle pressure waveform*, $P_{\text{cyc}}(x,t) = P - P_{\text{his,1}},$ can then be calculated; this is made up of wavefronts generated within the current cycle.

In addition, the contribution to the current cycle from the *n*th previous cycle can be calculated as $P_{\text{his,}n} - P_{\text{his,}n+1},$ where

16 $$\begin{aligned} P_{\text{his,}n}(t) = P_{\text{his,1}}(t + (n-1)T_{\text{c}}),{\quad t \ge T_{\text{c}}}. \end{aligned}$$

The history flow rate ($Q_{\text{his,n}}(x,t)$) driven by $P_{\text{his,n}} {- P_{\text{out}}}$ can be calculated as

17 $$\begin{aligned} Q_{\text{his,n}} = \frac{P_{\text{his,n}} - P_{\text{out}}}{R},\quad R = \frac{\overline{P} - P_{\text{out}}}{\overline{Q}}, \end{aligned}$$

where $\overline{P}$ is the mean pressure and $\overline{Q}$ the mean flow rate at the arbitrary point in the 1-D model network.

Using the peripheral–conduit separation described in “Conduit and Peripheral Waveforms” section, $P_{\text{cyc}}$ is decomposed into its peripheral component $P_{\text{cyc,per}} = P_{\text{per}} - P_{\text{his,1}},$ which is made up of wavefronts originating from reflections at terminal branches within the current cycle, and a component $P_{\text{cyc}} - P_{\text{cyc,per}}$ which is equal to the conduit pressure, $P_{\text{con}},$ and, therefore, made up of wavefronts propagating from the aortic root and their reflections at arterial junctions, tapered vessels, and the aortic root. Thus, $P$ is decomposed as18The flow waveform is decomposed into conduit, $Q_{\text{con}},$ and peripheral, $Q_{\text{per}},$ components associated with $P_{\text{con}}$ and $P_{\text{per}},$ respectively.

Lastly, $P_{\text{con}}$ and $Q_{\text{con}}$ are analyzed in two different ways. Wave intensity analysis (“Wave Intensity Analysis (WIA)” section) and separation into forward and backward components (“Forward- and Backward-Traveling Waveforms” section) applied to $P_{\text{con}}$ and $U_{\text{con}}$ allows us to calculate proximal ($P_{\text{con,f}},\,Q_{\text{con,f}}$) and distal ($P_{\text{con,b}},\,Q_{\text{con,b}}$) contributions to $P_{\text{con}}$ and $Q_{\text{con}}$:

19 $$\begin{aligned} P_{\text{con}} = P_{\text{con,f}} + P_{\text{con,b}}, \end{aligned}$$

20 $$\begin{aligned} Q_{\text{con}} = Q_{\text{con,f}} + Q_{\text{con,b}}, \end{aligned}$$

21 $$\begin{aligned} {\text{d}}I_{\text{con}}= {\text{d}}P_{\text{con}} \, {\text{d}}U_{\text{con}}. \end{aligned}$$

In addition, $P_{\text{con}}$ and $Q_{\text{con}}$ are separated into a *cardiac conduit waveform* ($P_{\text{con,c}},\,Q_{\text{con,c}}$) generated by the contraction of the left ventricle and a *vascular conduit waveform* ($P_{\text{con,v}},\,Q_{\text{con,v}}$) made up of reflected conduit wavefronts at arterial junctions and tapered vessels. They are calculated as

22 $$\begin{aligned} P_{\text{con,c}} = P_{\text{e,W3}},\quad P_{\text{con,v}} = P_{\text{con}} - P_{\text{con,c}}, \end{aligned}$$

23 $$\begin{aligned} Q_{\text{con,c}} = \frac{P_{\text{con,c}} }{Z_0},\quad Q_{\text{con,v}} = Q_{\text{con}} - Q_{\text{con,c}}, \end{aligned}$$

given the relationship between the pressures ($P_{\text{con}},\,P_{\text{per}}$) and ($P_{\text{e,W3}},\,P_{\text{r,W3}}$) discussed in “Reservoir and Excess Pressure Waveforms” section. $Q_{\text{con,c}}$ is the flow associated with $P_{\text{con,c}},$ since $P_{\text{con,c}}/Z_0$ is the conduit flow rate at an arbitrary location in a 1-D model network with well-matched arterial junctions for forward-traveling wavefronts and straight arterial segments, with $Z_0$ the local characteristic impedance.

## Results

We apply the methods of pulse wave analysis described in “Pulse Wave Analysis Methods” and “Combined Pulse Wave Analysis” sections to the pressure and flow waveforms simulated in the midpoint of the thoracic aorta, carotid, brachial and external iliac arteries (Figs. 1c and 1d) of the 55-artery model in normal conditions (“Normal Conditions—The ‘Healthy’ Model” section), with generalized arterial stiffening (“Generalized Arterial Stiffening—The ‘Old’ Model” section) and with localized changes in model parameters due to the presence of a carotid stent, femoral stenosis or abdominal aortic aneurysm (“Localized Changes in Model Parameters—Stenosis, Stent and Aneurysm” section).

### Normal Conditions—The ‘Healthy’ Model

Figure 1e compares the flow accelerations and forces per unit mass defined in Eq. (1b). The propagation of pulse waves at the start of the flow rise in early systole generates a pressure gradient force per unit mass (PG) that accelerates the flow first in the carotid artery (the site most proximal to the aortic root in Fig. 1), followed by the thoracic aorta, brachial and iliac arteries. Initially, the PG produces only temporal acceleration (TA). Convective acceleration (CA) dominates in the aorta in mid systole, but is negligible in more peripheral vessels at any time. Later in systole the flow is decelerated by the PG and viscous force per unit mass (VF). The VF is negative for most of the cardiac cycle and has a smaller magnitude in the aorta than in more peripheral sites. With increasing time in diastole, TA and CA tend to zero and PG to $-\text{VF}$ at any arterial site.

The wave intensity plots in Fig. 1f indicate that forward-traveling wavefronts dominate over backward-traveling wavefronts throughout systole. The flow is accelerated by a forward compression wave (FCW) in early systole, and decelerated by both a backward compression wave (BCW) in mid systole and a forward expansion wave (FEW) in late systole. FCWs have greater magnitudes than BCWs and FEWs in the four sites, and in the aorta, brachial and iliac arteries the FEW has a greater magnitude than the BCW; these two waves are similar in magnitude in the carotid artery. In late systole, TA (Fig. 1e) is produced by a FCW in the carotid and brachial arteries. In diastole wave intensity is zero in the scale of Fig. 1f.

Figures 1c and 1d show the forward- and backward-traveling components of the pressure and flow velocity waveforms. Forward pressure ($P_{\text{f}}$) and velocity ($U_{\text{f}}$) components dominate over backward components ($P_{\text{b}}$ and $U_{\text{b}}$) in systole. In early systole $P_{\text{f}}$ starts augmenting pressure before $P_{\text{b}}$ does. Similarly $U_{\text{f}}$ increases flow velocity before $U_{\text{b}}$ starts decreasing it, leading to reverse flow in the iliac artery. In late diastole, $P_{\text{f}}$ and $P_{\text{b}}$ converge to the same shape, as do $U_{\text{f}}$ and $-U_{\text{b}}.$

**Figure 3:**
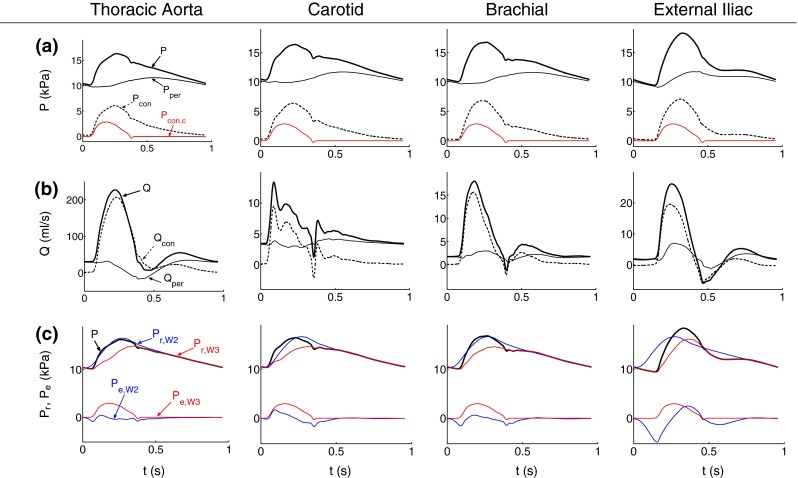
Pressure and flow waveforms in the midpoint of the thoracic aorta, left carotid, left brachial, and right external iliac of the 55-artery model sketched in Fig. 1a. (a) Pressure (P) and (b) flow (Q) waveforms separated into their peripheral (P$_{\text {per}},$ Q$_{\text {per}}$) and conduit components (P$_{\text {con}},$ Q$_{\text {con}}$). Cardiac conduit pressure (P$_{\text {con,c}}$) is plotted in red in (a). (c) Reservoir and excess pressures computed using the two-element ($\hbox {P}_{\text{r,W2}}, \hbox {P}_{\text{{e,W2}}}$) or three-element ($\text{P}_{\text{r,W3}}, \text{P}_{\text{e,W3}}$) Windkessel models

Figure 3a shows that peripheral pressure waveforms dominate over conduit waveforms throughout the cardiac cycle. Conduit pressures have a greater magnitude in systole than in diastole. They make up the main features of the pressure waveform in systole, such as most of the amplitude of the pressure pulse and the dicrotic notch or point of inflection at the end of systole; the notch completely disappears from peripheral pressure waveforms calculated using the linearized 1-D equations (1a), (1b), and (2) (data not shown). Figure 3b shows that conduit flows dominate over peripheral flows in systole and produce the main features of the flow waveform. Conduit flows vanish in diastole so that the flow is mainly peripheral towards the end of diastole.

Reservoir pressures $P_{\text{r,W2}}$ and $P_{\text{r,W3}}$ dominate over the excess pressures $P_{\text{e,W2}}$ and $P_{\text{e,W3}}$ throughout the cardiac cycle (Fig. 3c). $P_{\text{r,W2}}$ and $P_{\text{r,W3}}$ make up almost all the pressure waveform in diastole, when all pressures in the 1-D domain tend to approximately the same shape with increasing time. Excess pressures make up the main features of the pressure waveform in systole, in particular the dicrotic notch or point of inflexion. Lastly, note that $P_{\text{con}} \ge P_{\text{con,c}} = P_{\text{e,W3}}$ for the whole cardiac cycle (Fig. 3a). This result will be used below when quantifying the contribution to $P$ from reflections at tapered vessels and arterial junctions.

#### Combined Pulse Wave Analysis

**Figure 4:**
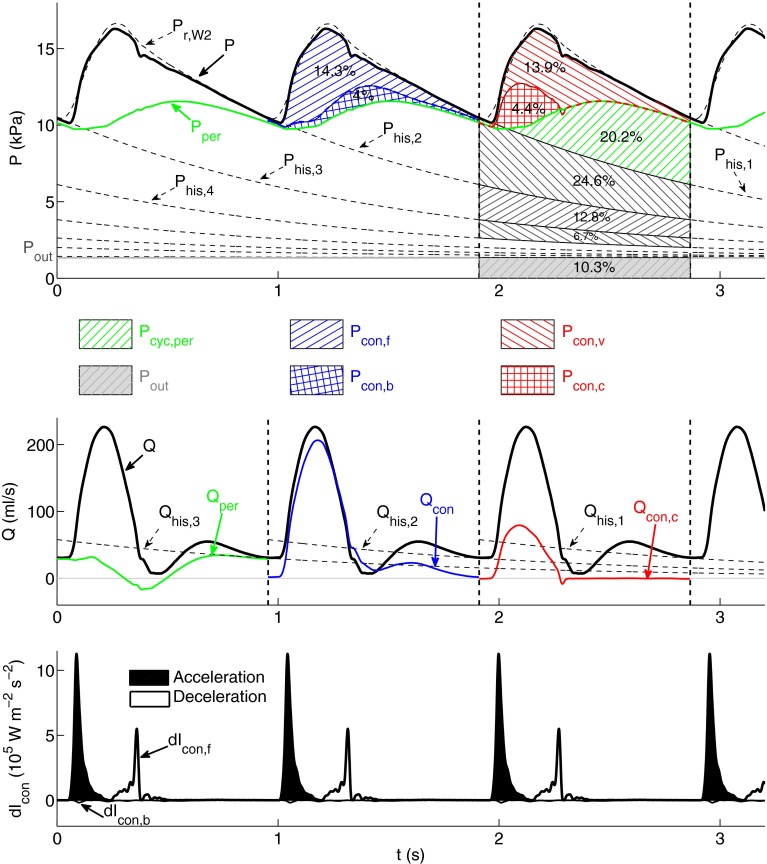
Analysis of the pressure ($P,$ top) and flow ($Q,$ middle) waveforms in the midpoint of the thoracic aorta of the ‘healthy’ model using the new methodology described in “Combined Pulse Wave Analysis” section. $P$ and $Q$ in the cardiac cycles bounded by dashed vertical lines are separated into different types of physical contributions. (top) Contributions to $P$ from forward- ($P_{\text{con,f}}$) and backward-traveling ($P_{\text{con,b}}$) conduit wavefronts (blue hatched area), vascular ($P_{\text{con,v}}$) and cardiac ($P_{\text{con,c}}$) conduit wavefronts (red hatched area), peripheral wavefronts originating within the current cardiac cycle ($P_{\text{cyc,per}},$ green hatched area), wavefronts originating within the three previous cardiac cycles which are calculated from the history pressures $P_{\text{his,1}},\,P_{\text{his,2}},\,P_{\text{his,3}}$ and $P_{\text{his,4}}$ (black hatched areas), and the outflow pressure $P_{\text {out}}$ (gray area). $P_{\text{per}}$ is the peripheral pressure. Contributions are quantified as a percentage of the total area under the pressure waveform. (middle) Peripheral ($Q_{\text{per}}$), conduit ($Q_{\text{con}}$), cardiac conduit ($Q_{\text{con,c}}$), and history ($Q_{\text{his,1}},\,Q_{\text{his,2}},\,Q_{\text{his,3}}$) flow waveforms. (bottom) Forward (${\text{d}}I_{\text{con,f}}$) and backward (${\text{d}}I_{\text{con,b}}$) components of conduit wave intensity (d$I_{\text{con}}$). Shaded waves (black) accelerate blood flow and non-shaded waves (white) decelerate blood flow

Using the combined wave analysis method we obtain that about 50% of the area under the pressure waveform in the thoracic aorta consists of history pressure ($P_{\text {his,1}}$; Fig. 4, top). The outflow pressure ($P_{\text{out}}$) contributes to this area with 10.3% and the remaining 38.5% is generated in the current cardiac cycle: 20.2% comes from ‘peripheral’ wavefronts originating from reflections at terminal branches and 18.3% from conduit reflections. These can be separated into (i) forward-traveling ‘conduit’ wavefronts (14.3%) and backward-traveling ‘conduit’ wavefronts (4.0%) or (ii) ‘cardiac’ wavefronts produced by the contraction of the left ventricle (4.4 %) and ‘vascular’ wavefronts reflected at tapered vessels and arterial junctions (13.9 %).

Figure 4 (middle) shows several types of flow waveforms in the thoracic aorta. The bulk of $Q$ is provided by the conduit component ($Q_{\text {con}}$). History ($Q_{\text {his,1}}$) and peripheral ($Q_{\text {per}}$) flows converge to the flow waveform ($Q$) with increasing time in diastole. Moreover, the cardiac conduit flow ($Q_{\text {con,c}}$) differs considerably from $Q_{\text {con}}$ in amplitude and shape, so that the vascular conduit flow ($Q_{\text {con,v}}$) produces about two thirds of the amplitude of $Q.$

The contributions of history pressures ($P_{\text {his,}n}$; Fig. 4, top) and their associated flows ($Q_{\text {his,}n}$; Fig. 4, middle) decrease exponentially with increasing number of previous cardiac cycles ($n$).

Wave intensity calculated from the conduit pressure and flow waveforms (Fig. 4, bottom) is very similar to traditional wave intensity calculated from $P$ and $U$ (Fig. 1f, thoracic aorta). In all the arteries studied we obtain a root mean square error (RMSE) <10% between these two wave intensity contours.

Combined pulse wave analysis in carotid, brachial and iliac arteries yields similar results to those described here for the thoracic aorta (see Figs. 1–3 in the supplementary material). The major difference is that in the carotid artery $Q_{\text {his,1}}$ does not converge to $Q$ with increasing time in diastole, but to a smaller flow rate (Fig. 1 in the supplementary material). Lastly we note that the combined analysis yields exactly the same results if $P_{\text{his,1}}$ is calculated by prolonging the exponential decay in $P_{\text{r,W3}}$ instead of that of $P_{\text{r,W2}}.$

### Generalized Arterial Stiffening—The *‘Old’* Model

**Figure 5:**
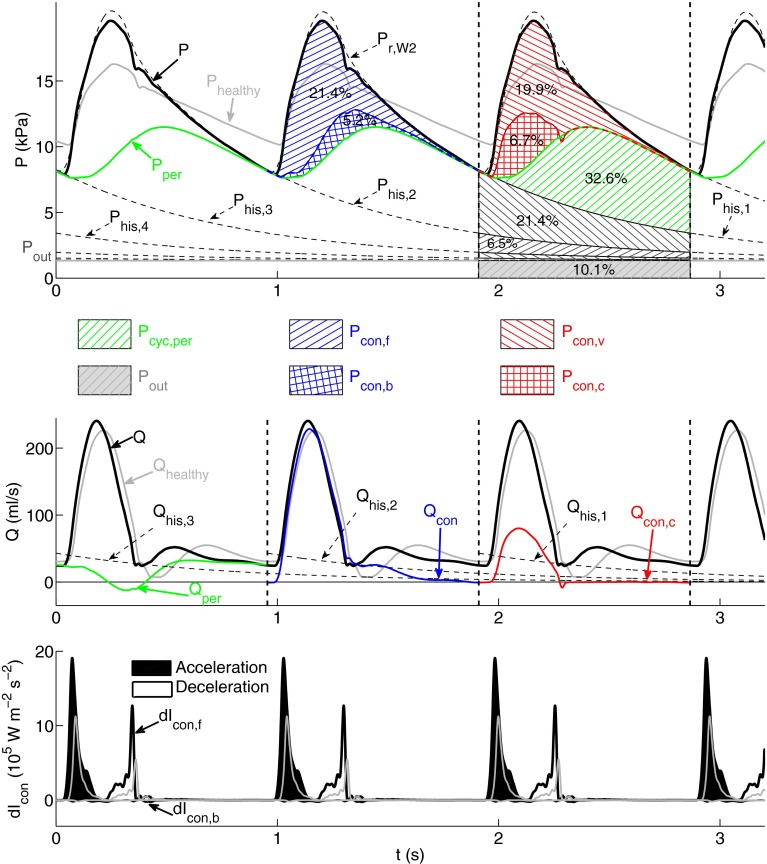
Analysis of the pressure ($P,$ top), flow ($Q,$ middle) and conduit wave intensity (d$I_{\text{con}},$ bottom) waveforms in the midpoint of the thoracic aorta of the ‘old’ model using the same format and methodology as described in Fig. 4. The corresponding total pressure ($P_{\text{healthy}}$), flow ($Q_{\text{healthy}}$) and conduit wave intensity waveforms for the ‘healthy’ model are superimposed in gray

The amplitude and mean value of the flow in the thoracic aorta of the ‘old’ model (Fig. 5, middle) are similar to those in the ‘healthy’ model (Fig. 4, middle). This is also the case for $Q_{\text {per}},\,Q_{\text {con}}$ and $Q_{\text {con,c}}$ (compare Figs. 4 and 5, middle). As in the ‘healthy’ model, $Q_{\text {per}}$ converges to $Q$ with increasing time in diastole. However, $Q_{\text {his,1}}$ produces only about 60% of $Q$ in end diastole instead of almost 100% in the ‘healthy’ model.

Greater variations in pressure and wave intensity waveforms are observed between the two models. The amplitudes of $P,\,P_{\text{con}},\,P_{\text{per}}$ (Fig. 5, top) and ${\text{d}}I_{\text{con}}$ (Fig. 5, bottom) are approximately doubled in the ‘old’ model. In addition, the cycle pressure waveform ($P_{\text{cyc}}$) accounts for about 60% of $P$ (38% in the ‘healthy’ model) due mainly to increased peripheral and vascular conduit contributions.

### Localized Changes in Model Parameters—Stenosis, Stent and Aneurysm

**Figure 6:**
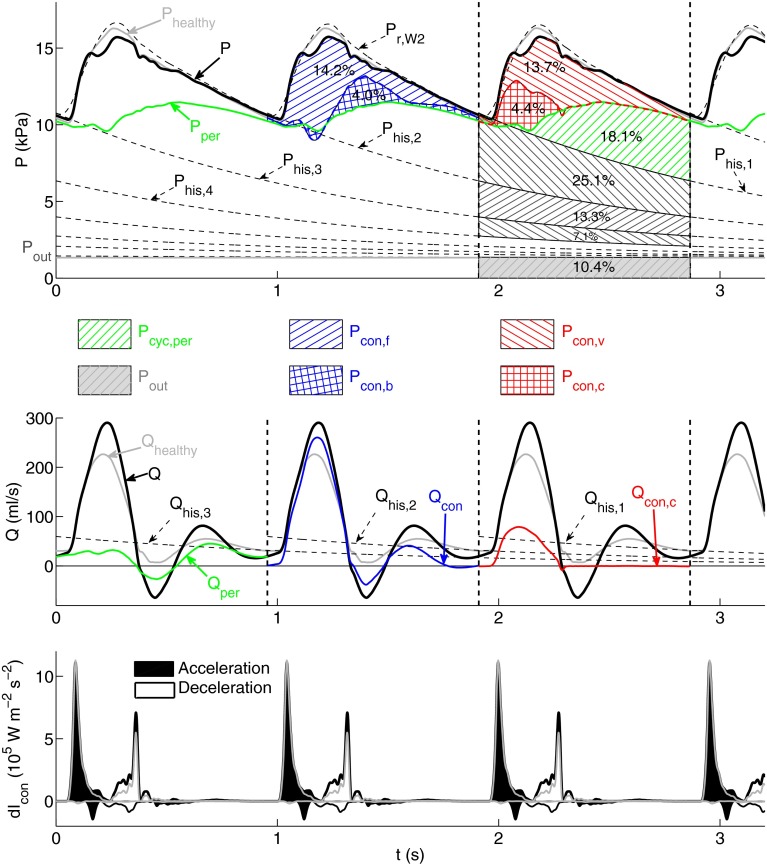
Analysis of the pressure ($P,$ top), flow ($Q,$ middle) and conduit wave intensity (d$I_{\text{con}},$ bottom) waveforms in the midpoint of the thoracic aorta of the model with an abdominal aortic aneurysm using the same format and methodology as described in Fig. 4. The corresponding total pressure ($P_{\text{healthy}}$), flow ($Q_{\text{healthy}}$) and conduit wave intensity waveforms for the ‘healthy’ model are superimposed in gray

The greatest changes in $P,\,Q$ and d$I$ due to a carotid stent, femoral stenosis or abdominal aortic aneurysm are observed in arterial segments near to where these three interventions are modeled, as shown in Fig. 4 in the supplementary material. Figure 6 shows the combined wave analysis applied to $P$ and $Q$ in the thoracic aorta with the downstream aneurysm. Combined analyses of $P$ and $Q$ upstream the stent and stenosis are shown, respectively, in Figs. 5 and 6 in the supplementary material. All three interventions produce different $P$ from the ‘healthy’ model due mainly to changes in wavefronts originating in the last cardiac cycle which make up $P_{\text{cyc}},$ rather than in wavefronts that make up the history pressure. Indeed, the time constant $R_{\text{T}} C_{\text{T}}$ that determines the history pressure in Eq. (10) changes little: it decreases by less than 1% with the stent, and increases by less than 1% with the stenosis and 4% with the aneurysm. The greatest variations in waveforms calculated by the combined wave analysis are observed in the conduit wave intensity of the stenosis and aneurysm. These include a threefold increase in the amplitude of the BCW upstream the stenosis (Fig. 6, bottom, in supplementary material), a tenfold increase in the amplitude of the BCW upstream the aneurysm (Fig. 6, bottom), and a threefold decrease in the amplitude of the FCW downstream the aneurysm (data not shown).

## Discussion

We have analyzed numerically generated pressure and flow waveforms using existing methods of pulse wave analysis and a new method that integrates the strengths of existing ones. Pulse wave analysis enables us to investigate the role of specific regions of the cardiovascular system on arterial hemodynamics, which is extremely challenging by direct comparison of forces and accelerations in the conservation of momentum Eq. (1b). Numerical data allows us to assess the strengths and limitations of all the studied methods without measurement errors and with complete knowledge of all the physical properties of the model. We discuss the physical information provided by existing methods of wave analysis (“Wave Intensity Analysis and Forward and Backward Waveforms”, “Peripheral and Conduit Waveforms” and “Reservoir and Excess Pressure Waveforms” sections) and our new one (“Combined Pulse Wave Analysis” and “Limitations of Combined Pulse Wave Analysis” sections).

### Wave Intensity Analysis and Forward and Backward Waveforms

Theoretically, wave intensity (d$I$) quantifies the importance of forward- and backward-traveling wavefronts throughout the cardiac cycle, and, hence, proximal and distal effects on arterial hemodynamics. In the aorta, carotid, brachial and iliac arteries, d$I$ contours contain three to four dominant waves in systole which propagate mostly in the forward direction and vanish in diastole (Fig. 1f), in agreement with *in vivo* data.^42^ According to WIA, therefore, pressure and flow waveforms in large arteries are mainly determined by wavefronts propagating from proximal to distal locations in systole. However, decomposition of pressure and flow waveforms into conduit and peripheral components (Figs. 3a and 3b) reveals that d$I$ fails to identify important contributions from wavefronts originating at peripheral reflection sites. Despite making up most of the pressure waveform throughout the cardiac cycle and the flow waveform in diastole, peripheral wavefronts produce much smaller changes in pressure (d$P$) and flow (d$U$) and, hence ${\text{d}} I = {\text{d}}P \, {\text{d}}U,$ than conduit contributions do. This limitation of WIA was discussed extensively in Alastruey *et al*.^3^

**Figure 7:**
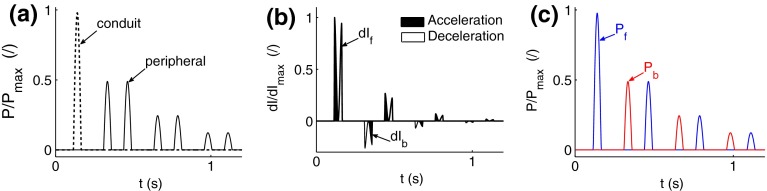
Pressure waveform at *x* = 0.4 m in a 1 m long straight tube with a uniform diameter of 2 cm and pulse wave velocity of 6.2 m s^−1^. A single pressure pulse with a narrow width is prescribed at the inlet as a reflective boundary condition and a reflection coefficient equal to 0.5 is prescribed at the outlet. (a) Conduit and peripheral components of pressure. (b) Forward (d*I*_f_) and backward (d*I*_b_) components of wave intensity. (c) Forward (*P*_f_) and backward (*P*_b_) components of pressure

Moreover, forward- and backward-traveling components of d$I,$ pressure and flow may be misleading indicators of the proximal (from the heart) or distal (from the periphery) origin of wavefronts. This is particularly important when quantifying cardiac and peripheral contributions to waveforms. Figure 7 illustrates this problem in a single-vessel with uniform properties and reflective boundaries. Propagation of a single pulse from the inlet generates multiple pulses reflected successively at the outlet (the periphery in this model) and inlet. The peripheral origin of reflected pulses is correctly identified by the conduit and peripheral components of pressure (Fig. 7a). However, reflected pulses originating at the outlet contribute to both d$I_{\text{f}}$ and $P_{\text{f}}$ once they are re-reflected at the inlet (Figs. 7b and 7c). Similarly, d$I_{\text{f}},\,P_{\text{f}}$ and $U_{\text{f}}$ in the thoracic aorta of the 55-artery model (Figs. 1c, 1d, and 1f) are made up of reflected wavefronts from peripheral branches in the upper body and re-reflections of reflected wavefronts originating at peripheral branches in the lower body. This is particularly clear in diastole, when pressure and flow waveforms are made up mostly of peripheral reflections (Figs. 3a and 3b), while forward- and backward-traveling pulse wavefronts yield similar contributions to pressure (Fig. 1c) and opposite contributions to flow velocity (Fig. 1d).

According to our numerical results, d$I$ and separation of pressure and flow into forward and backward components provide relevant physical information when analyzing the effect of a stenosis, stent or aneurysm. Nearby these interventions, d$I_{\text{f}}$ and d$I_{\text{b}}$ emphasize additional local reflections and provide changes in wave energy; this information is valuable to understand the separation into forward and backward components of pressure and flow waveforms. For example, Fig. 4 in the supplementary material shows that additional backward expansion waves are generated upstream the aortic aneurysm in systole, which is in agreement with the results reported in Swillens *et al*.^35^ These waves decrease $P$ producing a shoulder in the pressure waveform and accelerate the flow in mid systole leading to a greater flow amplitude than in the ‘healthy’ model.

### Peripheral and Conduit Waveforms

Separation into peripheral and conduit components (Fig. 3a and 3b) allows us to investigate the contribution to pressure and flow waveforms of wavefronts originating at the outlet of terminal branches or the rest of the arterial tree: the aortic root, arterial junctions, tapered vessels and other sites of impedance mismatch such as stenosis, stents and aneurysms. Conduit waveforms shape the main features of pressure and flow waveforms in systole, while peripheral waveforms play a particularly important role in shaping these waveforms in diastole, as discussed above.

Additional post-processing is required to isolate the effect of each reflection site (e.g., each arterial junction or terminal branch) on the pressure and flow waveforms. The ‘wave tracking’ algorithm proposed in Alastruey *et al*.^5^ enables us to do this for the conduit waveform in a uniform arterial tree. More research is required, however, to apply this algorithm to tapered arterial segments and to separate the contribution of each terminal branch to peripheral waveforms.

Separation into peripheral and conduit waveforms assumes conduit and peripheral wavefronts to add linearly. This is not completely satisfied when analyzing pressure and flow waveforms generated using the nonlinear 1-D equations (1a), (1b), and (2). As a result, nonlinearities affect the accuracy of conduit and peripheral waveforms. In particular, analysis of pressure and flow waveforms in the ‘healthy’ 55-artery model generated using the linearized 1-D equations yields smoother peripheral waveforms around the dicrotic notch, showing that the notch is entirely determined by conduit waveforms (data not shown), unlike in Fig. 3a.

### Reservoir and Excess Pressure Waveforms

The reservoir pressure calculated using the two- ($P_{\text{r,W2}}$) or three- ($P_{\text{r,W3}}$) element Windkessel model converges to the pressure waveform with increasing time in diastole (Fig. 3c), which is in agreement with *in vivo* results.^1^,^38^ We can describe the physical mechanism underlying this result using Eq. (10), which relates the reservoir pressure $P_{\text{r,W2}}$ to distributed 1-D model parameters: area and wave speed at mean pressure, arterial length, and peripheral compliances and resistances. Given that Eq. (10) neglects flow inertia and fluid viscous dissipation,^5^ we can conclude that both mechanisms vanish with increasing time in diastole. This observation is in agreement with decreasing temporal and convective accelerations of flow velocity and viscous forces with increasing time in diastole (Fig. 1e). In late diastole, therefore, the flow is driven by a decreasing pressure gradient, $P_{\text{r,W2}} - P_{\text{out}},$ that is space-independent and generated by the contraction of the arterial wall in all segments.

The excess pressure waveform $P_{\text{e,W2}}$ calculated from $P_{\text{r,W2}}$ does not seem to provide any relevant physical information. As for $P_{\text{e,W3}}$ and $P_{\text{r,W3}}$ we have previously shown that in an arterial tree with straight vessels and well-matched junctions for forward-traveling waves, $P_{\text{e,W3}} = P_{\text{con}}$ and $P_{\text{r,W3}} = P_{\text{per}}$.^2^ These equalities are not satisfied in our 55-artery models: $P_{\text{e,W3}} < P_{\text{con}}$ and $P_{\text{r,W3}} > P_{\text{per}}$ for most of the cardiac cycle in all arterial segments (see for example Figs. 3a and 3c). Wavefronts are, therefore, generated at ill-matched bifurcations and tapered vessels, leading to vascular conduit components of pressure, $P_{\text{con,v}} = P_{\text{con}} - P_{\text{con,c}},$ with $P_{\text{con,c}} = P_{\text{e,W3}}.$ Note that $P_{\text{con,c}}$ determines the dicrotic notch and vanishes at the start of diastole, which indicates that the diastolic pressure waveform is made up of wavefronts originating at both vascular conduit and peripheral reflection sites (Fig. 3a).

### Combined Pulse Wave Analysis

We have proposed a new approach to pulse wave analysis that integrates the methods discussed above to improve the post-processing of 1-D model results. Combined pulse wave analysis enables us to (i) quantify the buffering function of the aorta and other compliant vessels; (ii) quantify contributions to pressure and flow waveforms in a given cardiac cycle from wavefronts originating in previous cardiac cycles; and (iii) separate the contribution to pressure and flow in the current cardiac cycle into forward-traveling wavefronts from the aortic root, their reflections at peripheral reflection sites and their reflections at other sites of impedance mismatch such as tapered vessels and arterial junctions.

*(i) The buffering (or Windkessel) function of the aorta and other compliant vessels* decreases the amplitude of $P$ (the so called *pulse pressure*).^8^ $P_{\text{r,W2}}$ given by Eq. (10) provides a 0-D approximation to $P$ in systole at any large artery that can be used to quantify the contribution of each segment compliance to the buffering function of the arterial tree. We have seen that the buffering function is considerably reduced with arterial stiffening, but is not with the presence of a stent, stenosis or aneurysm. According to Eq. (10) this is due to larger changes in the time constant $R_{\text{T}} C_{\text{T}}$ of the diastolic decay in pressure with arterial stiffening: $R_{\text{T}} C_{\text{T}}$ decreases by 45% in the ‘old’ model, but changes by less than 4% with the stenosis, stent or aneurysm. According to Eqs. (12) and (13), the total arterial conduit compliance ($C_{\text{c}}$) and, hence $C_{\text{T}},$ decrease in the ‘old’ model due to greater pulse wave velocities $c_{\text{m}}(x)$ in all arterial segments leading to smaller distributed 1-D model compliances ($C^i_{\text{seg}},\,i=1, \ldots , N$).

*(ii) Contributions to* $P$ *from previous cardiac cycles* are quantified by history pressures ($P_{\text{his,n}}$) calculated using the reservoir pressure $P_{\text{r,W2}}$ (as defined by Eqs. (15) and (16)). We have seen that $P_{\text{his,1}}$ makes up about 50% of $P$ in the thoracic aorta, carotid, brachial and iliac arteries of the ‘healthy’ model (Fig. 4, top, and Figs. 1 to 3, top, in the supplementary material). This percentage changes by less than 2% in arteries near the simulated stenosis, stent or aneurysm, since the time constant $R_{\text{T}} C_{\text{T}}$ of $P_{\text{r,W2}}$ and, hence $P_{\text{his,n}},$ is changed by less than 4% relative to the ‘healthy’ model, as discussed above.

On the other hand, $P_{\text{his,n}}$ makes up less than 30% of $P$ with generalized arterial stiffening (Fig. 5, top) due to the decrease in $C_{\text{T}}$ and, hence $R_{\text{T}} C_{\text{T}},$ discussed above. Generalized arterial stiffening also reduces the magnitude of $Q_{\text{his,n}}$ driven by $P_{\text{his,n}} - P_{\text{out}},$ so that about half of end-diastolic $Q$ at the thoracic aorta is produced within the current cardiac cycle (Fig. 5, middle), instead of almost none in the ‘healthy’ model. Thus, cardiac contraction must generate a larger increase in pressure gradient within each cardiac cycle to drive blood flow in a stiffer network. This leads to an increase in the work done by the left ventricle to eject blood flow into the aorta, which may result in the ventricle to develop hypertrophy (a heart muscle disease) and to fail.^23^

In all cases studied, we have seen that wavefronts originating at peripheral reflection sites make up most of $P_{\text{his,1}},$ since $P_{\text {his,1}} < P_{\text {per}}$ for most of the cardiac cycle (e.g., see Figs. 4, 5 and 6, top). End-diastolic $Q$ is mainly determined by the peripheral waveform $Q_{\text {per}},$ with a considerable contribution from $Q_{\text{his,1}}$ (e.g., see Figs. 4, 5 and 6, middle). Earlier cycles contribute less to $P_{\text {his,1}}$ and $Q_{\text {his,1}}$ than later cycles do, since $P_{\text {his,n}}$ decays exponentially (Eqs. (15) and (16)). Peripheral wavefronts persist for several cardiac cycles because they are trapped in the arterial network between the aortic valve and terminal branches.^5^

*(iii) Contributions to* $P$ *in the current cardiac cycle* from wavefronts originating at peripheral reflection sites make up about 50% of $P_{\text {cyc}}$ in all models (see for example Figs. 4, 5 and 6, top). At the start of systolic ejection, most of $P$ is made up of peripheral wavefronts originating in previous cardiac cycles. Throughout systole, conduit wavefronts that make up $P_{\text {con}}$ are responsible for several features of $P,$ such as the dicrotic notch and pulse pressure. In diastole, however, $P_{\text {con}}$ decreases exponentially (Fig. 3a) and contributes little to $P$ in the next cardiac cycle. Therefore, $P_{\text {per}}$ provides most of the pressure gradient that drives the flow at the beginning and end of the cardiac cycle. Throughout the cardiac cycle, however, $Q_{\text {con}}$ is more similar in shape to $Q$ than $Q_{\text {per}},$ which suggests that $Q$ is mainly made up of conduit wavefronts (see Figs. 4, 5 and 6, middle).

Conduit waveforms allow us to investigate contributions to pressure and flow from proximal and distal reflection sites that are not peripheral. For example, comparison of Figs. 4 and 5 (top) reveals that the shape of the backward component of conduit $P$ ($P_{\text{con,b}}$) does not change significantly with generalized arterial stiffening. This result indicates that conduit reflections at distal sites are not increased. On the other hand, the conduit wave intensity contours (Figs. 4 and 5, bottom) highlight the increase in $P_{\text{con,f}}$ with arterial stiffening. Thus, conduit wave intensity (${\text{d}}I_{\text{con}}$) reveals whether forward or backward wavefronts are dominant. ${\text{d}}I_{\text{con}}$ is very similar to traditional wave intensity (${\text{d}}I$), which suggests that ${\text{d}}I$ quantifies the timing, direction and magnitude of conduit (rather than peripheral) wavefronts over the cardiac cycle.

In all the models studied, $P_{\text{con}}$ differs from $P_{\text{con,c}} = P_{\text{e,W3}}$ (Fig. 3a) The difference $P_{\text{con}} - P_{\text{e,W3}} = P_{\text{con,v}}$ is made up of reflections of wavefronts propagated by the left ventricle in the current cardiac cycle at arterial junctions, tapered vessels and other sites of impedance mismatch such as stenosis, stents and aneurysms. These vascular conduit wavefronts make up more than 70% of $P_{\text{con}}$ in all models studied, increasing considerably the pulse pressure and the amplitude of the flow waveform. Generalized arterial stiffening increases the amplitude of $P_{\text{con,c}}$ in any arterial segments, while localized changes in model properties introduced by stenosis, stents or aneurysms modify $Q_{\text{con,c}}$ locally. This is because stiffening of all arteries increases the pulse wave velocity at the aortic root and, hence, $Z_{\text{Ao}}$, which produces a greater amplitude in $P_{\text{con,c}}$ (see Eqs. 14 and 22), while the stenosis, stent or aneurysm only modifies pulse wave velocities locally, and hence the local $Z_0$ that determines $Q_{\text{con,c}}$ (Eq. 23), without changing $Z_{\text{Ao}}.$

Lastly, it is important to note that our combined pulse wave analysis can be theoretically applied to non-periodic waveforms, such as ectopic beats. Indeed, periodicity is not required to calculate any of the waveforms computed in the combined analysis. However, the use of our novel approach to non-periodic waveforms still needs to be explored.

### Limitations of Combined Pulse Wave Analysis

Combined pulse wave analysis neglects nonlinear effects to (i) calculate conduit and peripheral components, (ii) obtain $P_{\text{his,n}}$ using Eqs. (15) and (16), (iii) add forward- and backward-traveling conduit wavefronts, and (iv) separate conduit waveforms into cardiac and vascular components. In all the models studied, nonlinearities have a secondary effect on the simulated pressure and flow waveforms and, hence, the results from our novel analysis provide a reasonable first-order approximation to contributions from distinct parts of the 1-D domain. We have already discussed the effect of nonlinearities on the conduit and peripheral components of the dicrotic notch, and the ability of $P_{\text{r,W2}}$ to capture the diastolic decay in pressure for all simulations. However, nonlinear effects could become more relevant when analyzing waveforms with greater variations in pressure throughout the cardiac cycle than those discussed here. For example, the assumption of a constant $C_{\text{T}}$ to calculate $P_{\text{his,n}}$ may not be a reasonable one when analyzing ectopic beats and flow control mechanisms leading to big variations in pressure and, hence, $C_{\text{T}}.$

Application of our novel analysis to *in vivo* data is not straightforward. $P_{\text {his,n}}$ could be obtained *in vivo* by prolonging the decay in the measured pressure waveform from previous cardiac cycles,^3^ and hence without using Eqs. (15) and (16) which require knowledge of all 1-D model parameters. However, calculation of conduit and peripheral waveforms can be only done if all model parameters are known, which is very challenging *in vivo*. Recent studies have shown techniques to parametrise 1-D models in order to reproduce patient-specific *in vivo* data with some accuracy.^15^,^27^,^40^ These techniques offer the possibility to apply combined pulse wave analysis to a 1-D model that closely matches *in vivo* data in order to uncover the underlying physical mechanisms of *in vivo* waveforms. This approach, however, still needs to be fully explored.

## Conclusions

We have shown that existing methods of pressure and flow wave analysis provide complementary physical information that can be combined into a new method for improved analysis. The new method allows us to quantify contributions to numerically generated pressure and flow waveforms at an arbitrary arterial location from forward-traveling wavefronts generated by left ventricular contraction, from reflected wavefronts originating during previous cardiac cycles, from reflected wavefronts originating at peripheral reflection sites, or at tapered vessels and arterial junctions, and from the buffering function of the arterial tree. We have demonstrated the utility of our new method by post-processing waveforms generated in a distributed 1-D model under normal physiological conditions, with generalized arterial stiffening, or with the presence of a single stenosis, stent or aneurysm.

## Electronic supplementary material

Below is the link to the electronic supplementary material.

Electronic supplementary material 1 (PDF 1495 kb)
