# Supplementary material for: Arterial Pressure and Flow Wave Analysis Using Time-Domain 1-D Hemodynamics
Source: Ann Biomed Eng. 2014 Aug 20;43(1):190–206. doi: 10.1007/s10439-014-1087-4 (PMC4286649; doi:10.1007/s10439-014-1087-4)
Supplement: Supplementary file 1 — Electronic supplementary material 1 (PDF 1495 kb) [file 10439_2014_1087_MOESM1_ESM.pdf]

Annals of Biomedical Engineering

Arterial Pressure and Flow Wave Analysis Using Time-Domain 1-D Hemodynamics

Supplementary Material

Marie Willemet · Jordi Alastruey

We first show the results of the combined pulse wave analysis in the left carotid (Fig. 1), left brachial (Fig. 2) and right external iliac (Fig. 3) arteries of the 'healthy' model. We then compare pressure, flow velocity and wave intensity waveforms for the 'healthy' model and three clinical conditions considered in our study (Fig. 4): a stent in the internal carotid artery, a stenosis in the right femoral artery and an aneurysm in the abdominal aorta. Lastly, we show the results of the combined pulse wave analysis in the common carotid artery of the model with a stent (Fig. 5) and the iliac artery of the model with a stenosis (Fig. 6).

MW and JA gratefully acknowledge the support of an EPSRC project grant (EP/K031546/1). JA gratefully acknowledges the support of a British Heart Foundation Intermediate Basic Science Research Fellowship (FS/09/030/27812) and the Centre of Excellence in Medical Engineering funded by the Wellcome Trust and EPSRC under grant number WT 088641/Z/09/Z.

Address correspondence to J. Alastruey, Division of Imaging Sciences and Biomedical Engineering, St. Thomas' Hospital, King's College London, London, UK. Electronic mails: marie.willemet@kcl.ac.uk; jordi.alastruey-arimon@kcl.ac.uk

1 'Healthy' Model

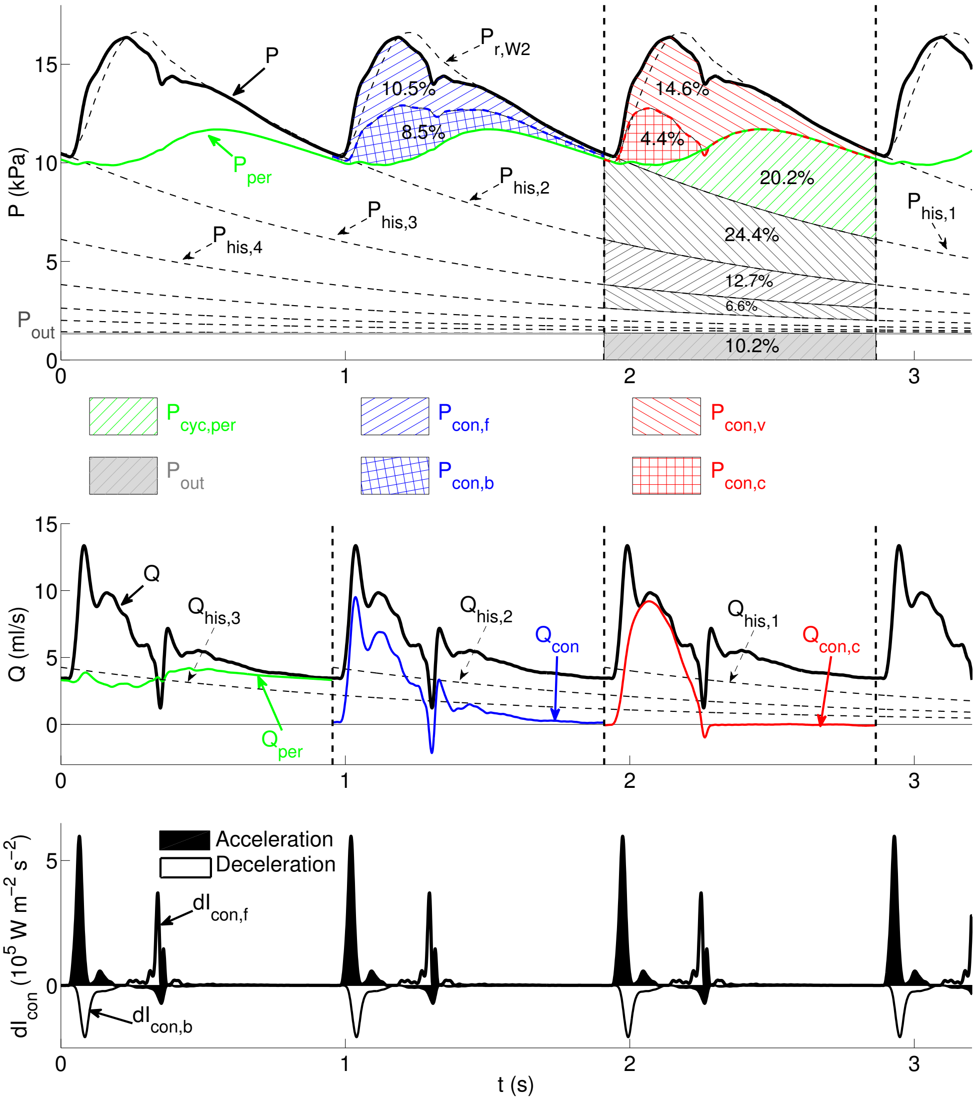

Fig. 1 Analysis of the pressure (P, top) and flow (Q, middle) waveforms in the midpoint of the left common carotid artery of the 'healthy' model using our new combined pulse wave analysis. P and Q in the cardiac cycles bounded by dashed vertical lines are separated into different types of physical contributions. (top) Contributions to P from forward- ($P_{\mathrm{con,f}}$) and backward-travelling ($P_{\mathrm{con,b}}$) conduit wavefronts (blue hatched area), vascular ($P_{\mathrm{con,v}}$) and cardiac ($P_{\mathrm{con,c}}$) conduit wavefronts (red hatched area), peripheral wavefronts originating within the current cardiac cycle ($P_{\mathrm{cyc,per}}$, green hatched area), wavefronts originating within the three previous cardiac cycles which are calculated from the history pressures $P_{\mathrm{his,1}}$, $P_{\mathrm{his,2}}$, $P_{\mathrm{his,3}}$ and $P_{\mathrm{his,4}}$ (black hatched areas), and the outflow pressure (P_{out}, gray area). P_{per} is the peripheral pressure. Contributions are quantified as a percentage of the total area under the pressure waveform. (middle) Peripheral (Q_{per}), conduit (Q_{con}), cardiac conduit ($Q_{\mathrm{con,c}}$) and history ($Q_{\mathrm{his,1}}$, $Q_{\mathrm{his,2}}$, $Q_{\mathrm{his,3}}$) flow waveforms. (bottom) Forward ($\mathrm{d}I_{\mathrm{con,f}}$) and backward ($\mathrm{d}I_{\mathrm{con,b}}$) components of conduit wave intensity ($\mathrm{d}I_{\mathrm{con}}$). Shaded waves (black) accelerate blood flow and non-shaded waves (white) decelerate blood flow.

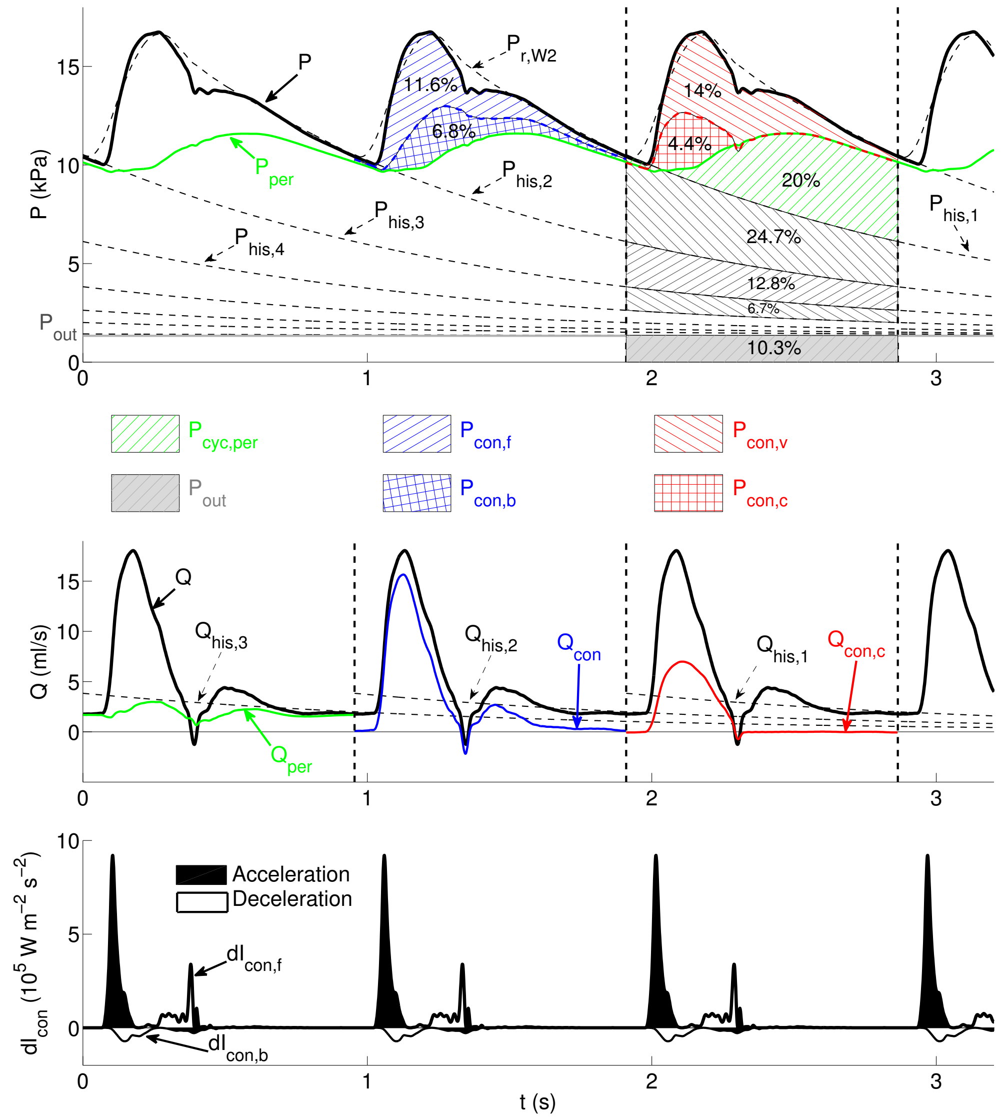

Fig. 2 Analysis of the pressure (P, top), flow (Q, middle) and conduit wave intensity ($\mathrm{d}I_{\mathrm{con}}$, bottom) waveforms in the midpoint of the left brachial artery of the 'healthy' model using the same format and methodology as described in Fig. 1.

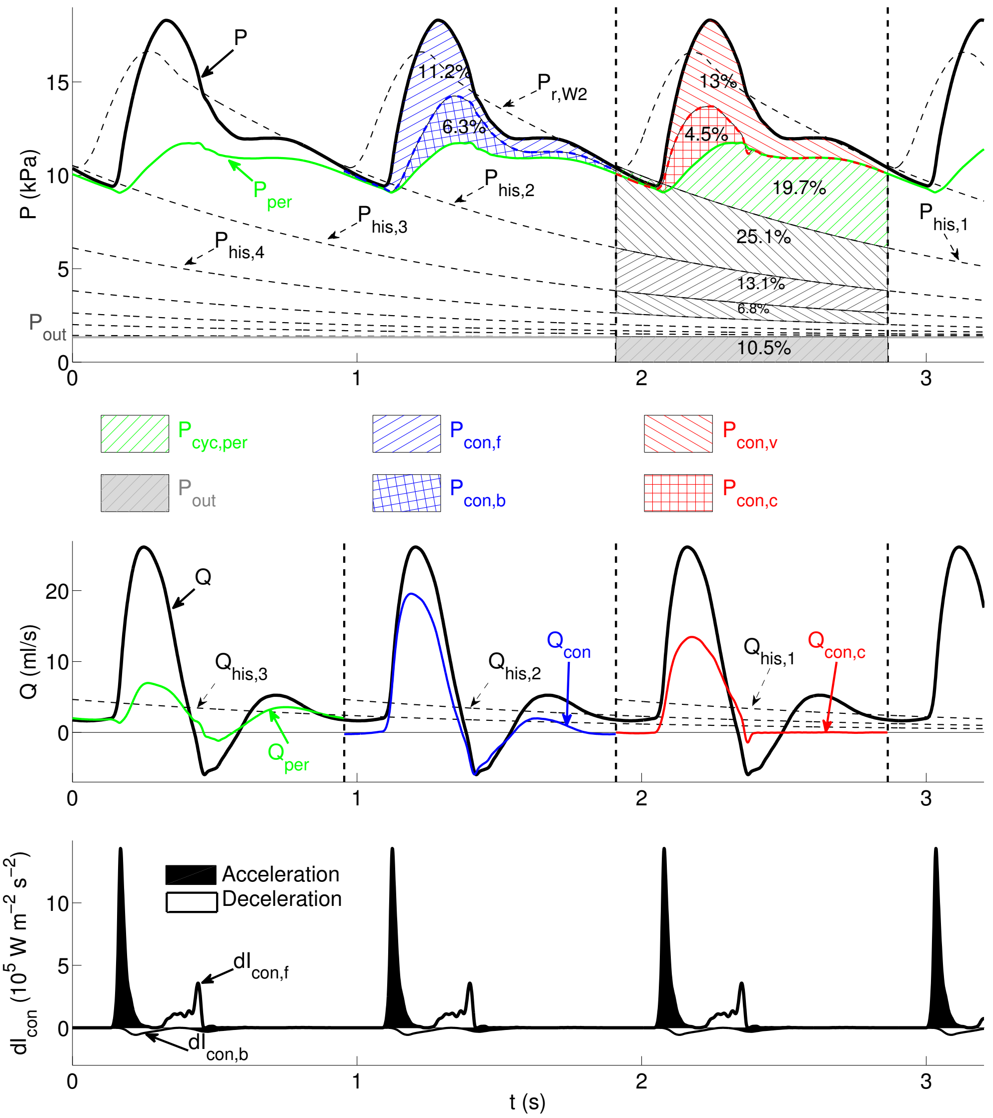

Fig. 3 Analysis of the pressure (P, top), flow (Q, middle) and conduit wave intensity (dI_{con}, bottom) waveforms in the midpoint of the right external iliac artery of the 'healthy' model using the same format and methodology as described in Fig. 1.

2 Clinical Conditions

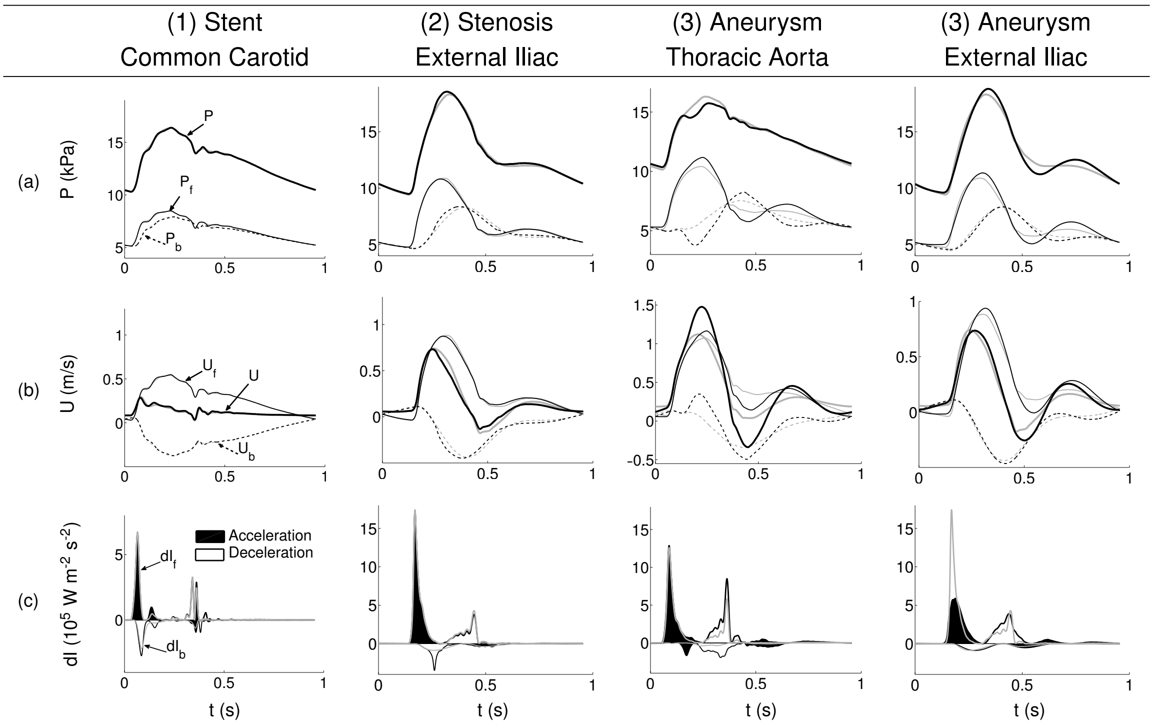

Fig. 4 Effects on (a) pressure (P, P_f, P_b), (b) velocity (U, U_f, U_b) and (c) wave intensity (dI_f, dI_b) waveforms (1) in the left common carotid artery of a stent in the left internal carotid artery; (2) in the right external iliac artery of a stenosis in the right femoral artery; and (3) in the thoracic aorta and external iliac artery of an aneurysm in the abdominal aorta. Waveforms in black refer to the model with stent, stenosis or aneurysm, and waveforms in gray refer to the 'healthy' model.

2.1 Internal Carotid Artery Stent

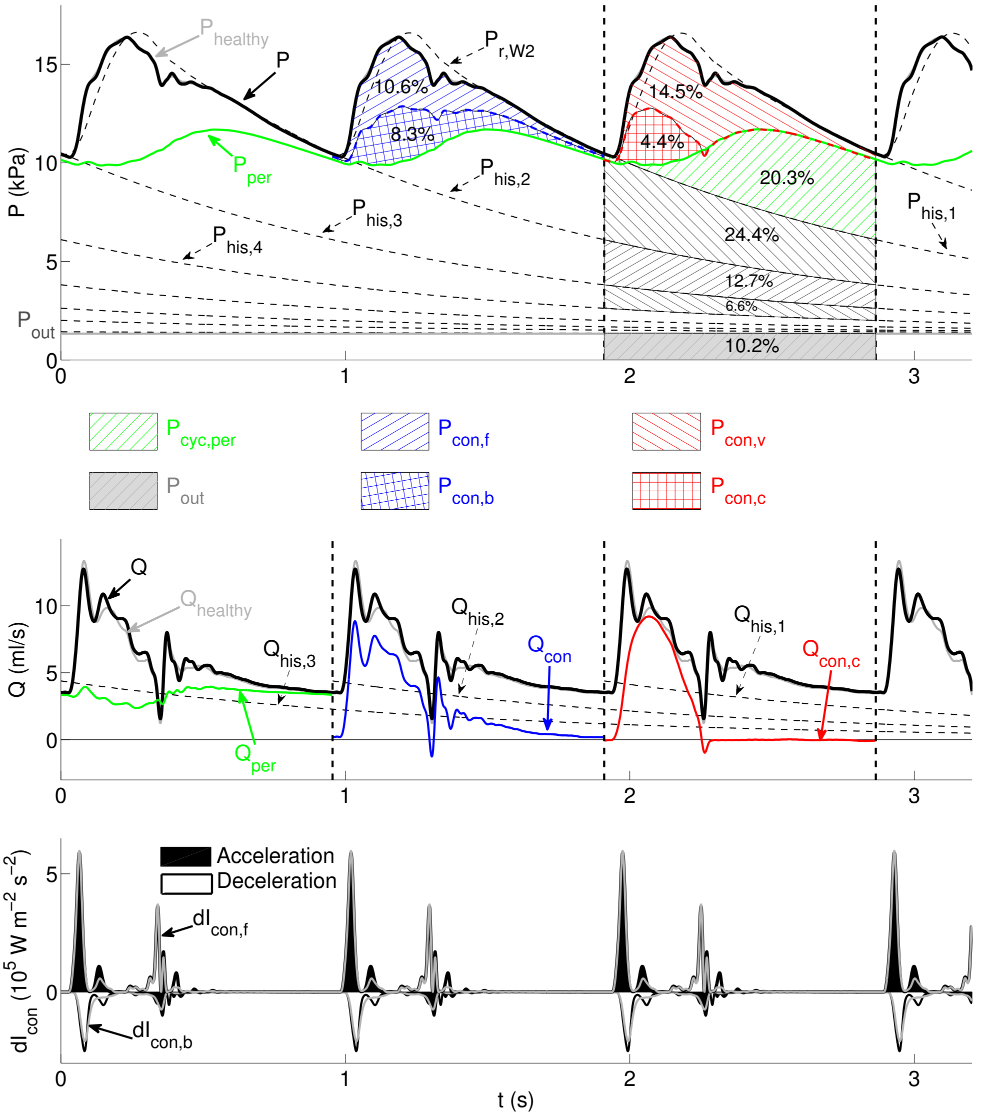

Fig. 5 Analysis of the pressure (P, top), flow (Q, middle) and conduit wave intensity ($\mathrm{d}I_{\mathrm{con}}$, bottom) waveforms in the midpoint of the common carotid artery of the model with a stent in the proximal left internal carotid artery using the same format and methodology as described in Fig. 1. The corresponding total pressure (P_{healthy}), flow (Q_{healthy}) and conduit wave intensity waveforms for the 'healthy' model are superimposed in gray.

2.2 Femoral Artery Stenosis

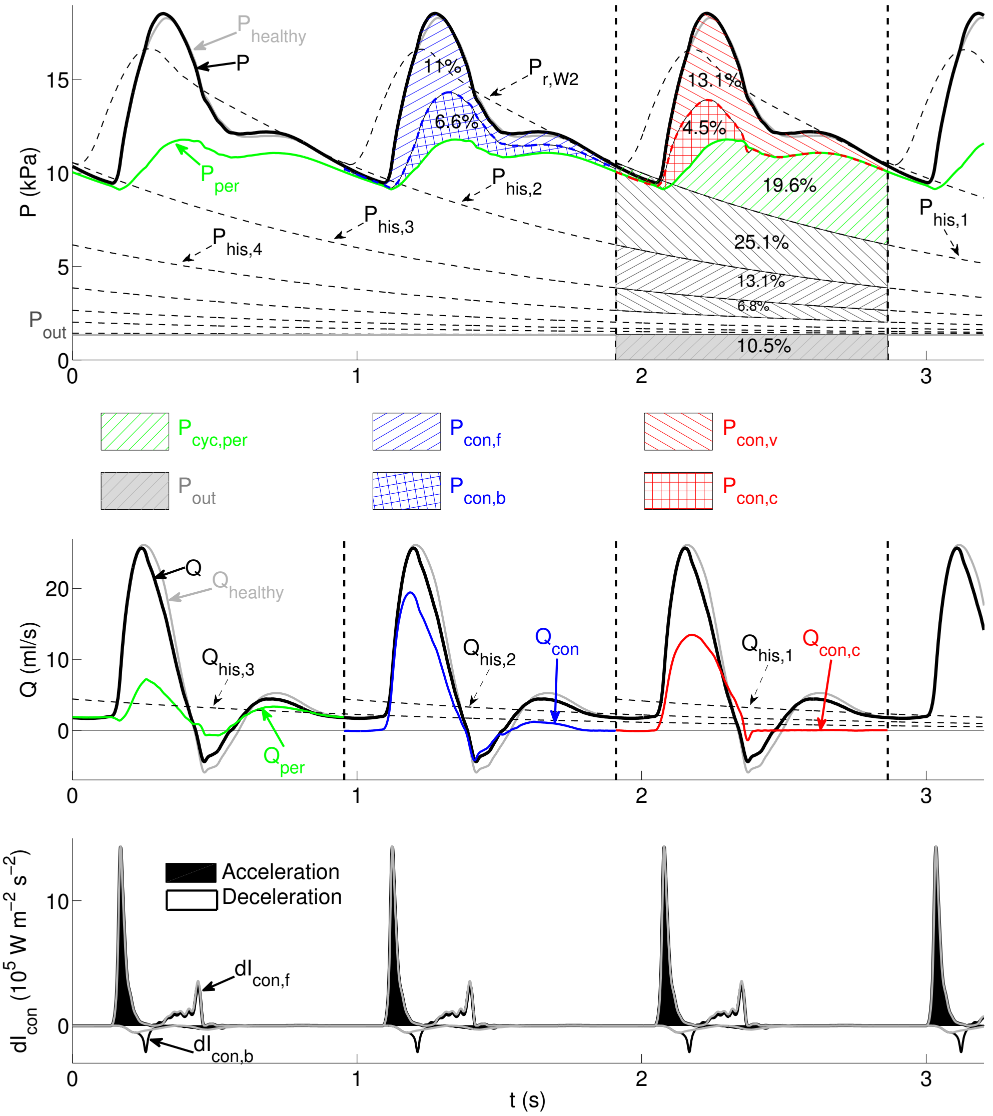

Fig. 6 Analysis of the pressure (P, top), flow (Q, middle) and conduit wave intensity ($\mathrm{d}I_{\mathrm{con}}$, bottom) waveforms in the midpoint of the right iliac artery of the model with a stenosis in the midpoint of the right femoral artery using the same format and methodology as described in Fig. 1. The corresponding total pressure (P_{healthy}), flow (Q_{healthy}) and conduit wave intensity waveforms for the 'healthy' model are superimposed in gray.
